# Comparison of sedation with pentazocine or pethidine hydrochloride for endoscopic ultrasonography in outpatients: A single‐center retrospective study

**DOI:** 10.1002/deo2.70048

**Published:** 2024-12-31

**Authors:** Makiko Urabe, Kenji Ikezawa, Yusuke Seiki, Ko Watsuji, Yasuharu Kawamoto, Takeru Hirao, Yugo Kai, Ryoji Takada, Takuo Yamai, Kaori Mukai, Tasuku Nakabori, Hiroyuki Uehara, Kazuyoshi Ohkawa

**Affiliations:** ^1^ Department of Hepatobiliary and Pancreatic Oncology Osaka International Cancer Institute Osaka Japan

**Keywords:** EUS, midazolam, pentazocine, pethidine hydrochloride, sedation

## Abstract

**Objectives:**

Endoscopic ultrasonography (EUS) plays an important role in the diagnosis of pancreatobiliary diseases. However, an appropriate sedation method for EUS has not been established. Therefore, this study aimed to examine the safety and complications of sedation with pentazocine or pethidine hydrochloride for outpatient diagnostic EUS.

**Methods:**

We retrospectively reviewed 1302 consecutive cases in our department that underwent outpatient diagnostic EUS between April 2019 and September 2021. Until June 2020, EUS was performed under sedation with midazolam and pentazocine (pentazocine group) in principle; after June 2020, sedation with midazolam and pethidine hydrochloride (pethidine hydrochloride group) was used. A cohort of patients with comparable backgrounds was identified using propensity score matching.

**Results:**

A total of 486 cases were included in this study. Sedation‐related adverse events during the endoscopic procedures were not significantly different between the groups. The median time spent in the recovery room after EUS was significantly shorter in the pethidine hydrochloride group than in the pentazocine group (69 versus vs. 77 min; *p *< 0.001). The frequency of nausea or vomiting after EUS was significantly lower in the pethidine hydrochloride group than in the pentazocine group (0% [0/486] vs. 6.2% [29/486]; *p *< 0.001). The frequency of readmission to the recovery room after discharge was significantly lower in the pethidine group than in the pentazocine group (0 [0%] vs. 18 [3.7%], respectively; *p *< 0.001).

**Conclusions:**

The combination of midazolam and pethidine hydrochloride is a more favorable anesthetic than the combination of midazolam and pentazocine for diagnostic EUS in outpatients.

## INTRODUCTION

In recent years, the number of patients with pancreatic and biliary tract cancer has been increasing.^1^, ^2^ Pancreatic cancer has a poor prognosis because early diagnosis of pancreatic cancer is often difficult with conventional diagnostic imaging modalities, e.g., computed tomography and magnetic resonance imaging.^3^, ^4^ Endoscopic ultrasonography (EUS) can detect small pancreatic cancer without the use of ionizing radiation or contrast agents.^5^, ^6^ EUS observation using new techniques, e.g., elastography and detective flow imaging, enhances the diagnostic capabilities of EUS^7^, ^8^ and plays an important role in the surveillance of pancreatic cancer.^9^, ^10^ In patients with biliary tract cancer, EUS provides valuable information regarding the extent of the cancer and lymph node metastasis.^11^ EUS is also useful in the differentiation between malignant and benign biliary strictures.^12^ Thus, the importance of EUS for pancreatobiliary diseases has been increasing.

Sedated endoscopy has improved patient acceptance and satisfaction and has contributed to the improvement of diagnostic procedure quality and treatment outcomes.^13^, ^14^, ^15^, ^16^ The optimal level of sedation for endoscopic procedures is moderate, which allows patients to maintain ventilatory and cardiovascular functions and to respond purposefully to verbal or light tactile stimulation.^14^, ^16^ Sedation lowers the level of consciousness, whereas analgesia reduces pain without lowering the level of consciousness.^15^ If sedatives alone do not provide adequate sedation, a combination of sedatives and analgesics may be effective.^13^, ^15^ According to national survey reports in Japan, the most commonly used sedative is midazolam, and the most commonly used analgesics are pentazocine and pethidine hydrochloride.^17^ The combination of midazolam and pentazocine is the conventional method used in Japan, but few studies have compared sedation with pentazocine to sedation with other analgesics.^15^, ^18^ In this study, we retrospectively examined the safety and complications of sedation with pentazocine or pethidine hydrochloride for diagnostic EUS in outpatients.

## METHODS

### Study design and patients

We retrospectively reviewed 1302 consecutive cases who underwent diagnostic EUS in an outpatient setting at our hospital between April 2019 and September 2021. Until June 2020, in principle, the endoscopy was performed under sedation with a combination of midazolam and pentazocine; after June 2020, sedation with midazolam and pethidine hydrochloride was used. Pentazocine or pethidine hydrochloride was administered intravenously in addition to midazolam at the initiation of endoscopy.

This study was approved by the Institutional Review Board of Osaka International Cancer Institute (approval number: 20117‐6) and was designed and executed in accordance with the ethical standards of the 1964 Declaration of Helsinki and its later amendments.

### EUS procedures, monitoring, and recovery

All EUS procedures were performed by endoscopists in our department using a linear‐array echoendoscope (GF‐UCT260; Olympus Medical Systems). After the initial administration of sedatives and analgesics by endoscopists, endoscope insertion was started when the endoscopist judged the Ramsey Sedation scale score as 5 or 6. The addition of sedatives or analgesics was performed by endoscopists or nurses when deemed necessary by endoscopists to maintain appropriate sedation. Each endoscopist determined the doses of midazolam, pentazocine, and pethidine hydrochloride. The patients received 2 L/min (min) oxygen via a nasal cannula from procedure initiation, and their vital signs were recorded and monitored every 5 min during the procedure. Oxygen saturation was recorded using a pulse oximeter (Nihon Kohden Co.). Cardiac function was measured using electrocardiography, and blood pressure was measured using an automatic inflatable cuff (Nihon Kohden Co.). Nurses recorded the procedure time (defined as the period between endoscope insertion and extubation), amount of medication, and sedation‐related adverse events during endoscopic procedures. Respiratory complications were defined as an oxygen saturation level less than 90%, requiring supplemental oxygen. Circulatory complications were defined as a decrease in systolic blood pressure below 90 mmHg, requiring the administration of intravenous fluids. Discontinuation of EUS was defined as cases where the endoscopists judged it unsafe or inappropriate to proceed. The use of antagonistic agent (flumazenil) was decided at the discretion of each endoscopist, such as in cases of reduced patient arousal. After EUS was completed, the patients were moved from the endoscopy room to the recovery room, which was part of the endoscopy suite, and they continued to be monitored. The discharge from the recovery room was determined by nurses based on an assessment of the anesthesia recovery criteria in our department. The anesthesia recovery criteria require that (1) the patient is able to say his name in response to calls; (2) the patient is able to take deep breaths and cough; (3) the patient can maintain a saturation of peripheral oxygen of ≥92% without oxygen; (4) the patient has a systolic blood pressure of ≥90 mmHg; (5) the patient has a heart rate of ≥50 bpm; (6) the patient has a healthy complexion and no nausea or vomiting; (7) at least 45 min have passed since the endoscopy extubation; (8) the patient can move his/her limbs freely and can walk independently without staggering. If all eight discharge criteria were met, the patient was judged to be ready for discharge. The time spent in the recovery room was calculated as the time from admission to the recovery room to discharge from the recovery room. The frequency of readmission to the recovery room after discharge from the recovery room was also calculated.

### Statistical analysis

Categorical variables are expressed as percentages, whereas continuous variables are presented as medians and interquartile ranges (IQRs). Baseline characteristics (patient sex, patient age, patient body mass index (BMI), performance status, reasons for diagnostic EUS, and EUS operator), procedure‐related outcomes (procedure time, doses of midazolam, and sedation‐related adverse events), duration of stay in the recovery room, nausea or vomiting after EUS, and readmission to the recovery room were compared using chi‐square and Fisher exact tests for categorical variables or the Mann–Whitney U‐test for continuous variables. The EUS operators were classified as experts and trainees. Experts were defined as endoscopists who had performed EUS for >3 years.

Propensity score matching was performed to identify patients with similar baseline characteristics. Age, sex, BMI, and operator of EUS were used as factors in the propensity score using logistic regression analysis. Briefly, a 1:1 matching protocol without replacement using a greedy matching algorithm was used with the caliper width set at 0.2 of the standard deviation of the logit of the propensity score. Standardized differences for each baseline covariate were calculated to evaluate balance before and after matching. Small imbalances were indicated by normalized differences of <0.10 for each covariate.^19^ To examine factors associated with readmission to the recovery room, odds ratios (ORs) and 95% confidence intervals (CIs) were calculated using univariate and multivariate logistic regression analyses. Factors with a *p*‐value <0.20 on univariate analysis were entered into the multivariate analyses. EZR (Saitama Medical Center, Jichi Medical University), a graphical interface for R, and R Commander software package for Windows (version 1.54) were used to perform all statistical analyses.^20^ Statistical significance was set at *p *< 0.05.

## RESULTS

### Baseline characteristics

A total of 1302 diagnostic EUS procedures were performed in our endoscopy unit during the study period. Pentazocine was used as an analgesic in 535 cases (pentazocine group), and pethidine hydrochloride was used in 767 cases (pethidine hydrochloride group). The baseline characteristics of the study population are summarized in Table 1. Before performing the propensity score‐matched analyses, we found marked differences in sex and EUS operators between the groups. After propensity score‐matched analyses, 486 patients were included in each group, and there were no significant differences in any of the variables in Table 2. The standardized differences in age, sex, BMI, and EUS operator were <0.1 after propensity score‐matched analyses.

**TABLE 1:** Baseline characteristics.

	Pentazocine *N* = 535	Pethidine hydrochloride *N* = 767
Sex, *n* (%)		
Male	347 (64.9)	330 (43.0)
Female	188 (35.1)	437 (57.0)
Age, years, median (IQR),	69 (60–74)	69 (59–73)
BMI, kg/m^2^, median (IQR)	22.5 (20.6–25.1)	22.5 (20.4–25.0)
Performance status, *n* (%)		
0 or 1	535	766
2<	0	1
Reason for diagnostic EUS, *n* (%)		
Pancreatic tumor	201 (37.6)	303 (39.5)
Intraductal papillary mucinous neoplasm	138 (25.8)	200 (26.0)
Pancreatic cysts	121 (22.6)	176 (23.0)
Pancreatic neuroendocrine tumor	13 (2.4)	16 (2.1)
Papillary tumor	32 (6.0)	37 (4.8)
Biliary tract tumor	13 (2.4)	16 (2.1)
Gallbladder tumor	7 (1.3)	12 (1.6)
Intraabdominal tumor	3 (0.6)	3 (0.4)
Duodenal tumor	1 (0.2)	0 (0)
Lymph node	6 (1.1)	2 (0.3)
Common bile duct stone	0 (0)	2 (0.3)
Operator of EUS, *n* (%)		
Expert	228 (42.6)	267 (34.8)
Trainee	307 (57.4)	500 (65.2)

Abbreviations: EUS, endoscopic ultrasonography; IQR, interquartile range.

**TABLE 2:** Baseline characteristics before and after propensity score matching.

	Original Data Set				Matched Data Set			
	Pentazocine *N* = 535	Pethidine hydrochloride *N* = 767	SMD	*P*‐value	Pentazocine *N* = 486	Pethidine hydrochloride *N* = 486	SMD	*P*‐value
Sex, n (%)								
Male	347 (64.9)	330 (43.0)	0.449	<0.001	300 (61.7)	300 (61.7)	<0.001	1.000
Female	188 (35.1)	437 (57.0)			186 (38.3)	186 (38.3)		
Age, years, median (IQR)	69 (60–74)	69 (59–73)	0.033	0.554	69 (60–74)	68 (59–73)	0.062	0.337
BMI, kg/m^2^, median (IQR)	22.5 (20.6–27.1)	22.5 (20.4–25.0)	0.087	0.126	22.5 (20.7–25.1)	22.9 (20.8–25.0)	0.003	0.968
Operator of EUS, n (%)								
Expert	228 (54.6)	267 (34.8)	0.161	0.005	186 (38.3)	186 (38.3)	<0.001	1.000
Trainee	307 (57.4)	500 (65.2)			300 (61.7)	300 (61.7)		

Abbreviations: BMI, body mass index; EUS, endoscopic ultrasonography; IQR, interquartile range; SMD, standardized mean difference.

### Procedure‐related outcomes

The procedure‐related outcomes of this study after propensity score matching are summarized in Table 3. The procedure time was not significantly different between the groups (median [IQR], pethidine hydrochloride group: 23 min [18–29 min] vs. pentazocine group: 22 min [16–28 min]; *p *= 0.072). The median pre‐EUS dose of pentazocine was 15 mg, whereas the median pre‐EUS dose of pethidine hydrochloride was 35 mg. The median pre‐EUS dose of midazolam was significantly lower in the pethidine hydrochloride group than in the pentazocine group (median [IQR], 0.050 mg/kg [0.043–0.061 mg/kg] vs. 0.058 mg/kg [0.048–0.069 mg/kg]; *p *< 0.001). The median total dose of midazolam was significantly lower in the pethidine hydrochloride group than in the pentazocine group (median [IQR], 0.068 mg/kg [0.057–0.080 mg/kg] vs. 0.075 mg/kg [0.061–0.095 mg/kg]; *p *< 0.001). There were no deaths or serious complications that required life‐saving treatment during the present study period. Although one patient in the pethidine hydrochloride group discontinued EUS because of respiratory depression, there was no significant difference in the frequency of sedation‐related adverse events during the endoscopic procedures between the groups. The frequency of administration of flumazenil after the endoscopic procedures did not differ significantly between the groups (pethidine hydrochloride group: 33 cases (6.7%) vs. pentazocine group: 32 cases (6.6%); *p *= 0.898).

**TABLE 3:** Procedure‐related outcomes (after propensity score matching).

	Pentazocine *N* = 486	Pethidine hydrochloride *N* = 486	*p*‐value
Procedure time, minute, median (IQR)	22 (16–28)	23 (18–29)	0.072
Pre‐EUS dose of analgesics, mg, median (IQR)	15 (15‐15)	35 (35‐35)	
Total dose of analgesics, mg, median (IQR)	15 (15‐15)	35 (35‐35)	
Pre‐EUS dose of midazolam, mg/body weight, median (IQR)	0.058 (0.048–0.069)	0.050 (0.043–0.061)	<0.001
Total dose of midazolam, mg/body weight, median (IQR)	0.075 (0.061–0.095)	0.068 (0.057–0.081)	<0.001
Administration of flumazenil, *n* (%)	32 (6.6)	33 (6.7)	0.898^*^
Sedation‐related adverse events during EUS, *n* (%)			
Discontinuation of EUS	0 (0.0)	1 (0.2)	1.000^**^
Circulation	9 (1.9)	3 (0.6)	0.146^**^
Respiration	4 (0.8)	2 (0.4)	0.682^**^

Abbreviations: EUS, endoscopic ultrasonography; IQR, interquartile range.

Statistical significance was set at *p *< 0.05.

Mann–Whitney U‐test.

* Chi‐square test.

** Fisher's exact test.

### Sedation‐related outcomes after EUS

The duration of stay in the recovery room was significantly shorter in the pethidine hydrochloride group than in the pentazocine group (median [IQR], 69 min [61–81 min] vs. 77 min [65–94 min]; *p *< 0.001; Table 4). The frequency of nausea or vomiting after EUS was significantly lower in the pethidine hydrochloride group than in the pentazocine group (0% [0/486] vs. 6.2% [29/486]; *p *< 0.001). In addition, the frequency of readmission to the recovery room was significantly lower in the pethidine hydrochloride group than in the pentazocine group (0% [0/486] vs. 3.7% [18/486]; *p *< 0.001). All the cases who were readmitted to the recovery room complained of nausea or vomiting, and three cases also complained of dizziness. In one case, the patient was hospitalized for persistent nausea and could not return home on the day of EUS. Finally, the multivariate analysis to examine factors associated with readmission showed that pentazocine (OR, 17.682; 95% CI, 6.066–51.542; *p *< 0.001) and female sex (OR, 8.220; 95% CI, 3.482–19.404; *p *< 0.001) were identified as independent factors (Table S1).

**TABLE 4:** Sedation‐related outcomes after endoscopic ultrasonography (after propensity score matching).

	Pentazocine *N* = 486	Pethidine hydrochloride *N* = 486	*p*‐value
Duration of stay in the recovery room, minutes, median (IQR)	77 (65–94)	69 (61–81)	<0.001
Nausea or vomiting after EUS, *n* (%)	29 (6.2)	0 (0)	<0.001
Readmission to the recovery room, n (%)	18 (3.7)	0 (0)	<0.001

Abbreviations: EUS, endoscopic ultrasonography; IQR, interquartile range.

Statistical significance was set at *p* < 0.05.

Mann–Whitney U‐test.

## DISCUSSION

Sedation methods that provide the sedation levels required for diagnostic EUS have not been well studied.^13^, ^15^ There is no specific evidence to support the level of sedation required for diagnostic EUS. This EUS is often performed in an outpatient setting because it is quicker to perform and is usually better tolerated than therapeutic EUS or endoscopic retrograde cholangiopancreatography. Moderate sedation is the most common practice when performing diagnostic EUS,^13^ and it is most often achieved through intravenous bolus delivery of benzodiazepines and opioid analgesics.^14^, ^21^ The combination of sedatives and opioid analgesics provides satisfactory moderate sedation because opioid analgesics alone provide mild sedation with analgesia.^13^, ^16^ However, published data have suggested that a combination of sedatives and opioid analgesics may increase the likelihood of sedation‐related adverse events.^22^, ^23^, ^24^ Several sedation‐related adverse events have been reported, including hypotension, desaturation, bradycardia, hypertension, respiratory depression, vomiting, and cardiac and respiratory arrest.^21^, ^25^ In a review of 40 patients who underwent EUS with a combination of midazolam and pethidine hydrochloride, no serious complications occurred; however, minor complications, such as hypoxemia, occurred in 51% of cases.^26^ When midazolam and pentazocine were used during radiofrequency ablation, airway insertion was required in 18% of patients.^27^ Here, sedation‐related adverse events during diagnostic EUS were < 2% in both the pentazocine and pethidine hydrochloride groups. However, the pentazocine group required a considerably longer time to recover from sedation than the pethidine hydrochloride group. Additionally, the pentazocine group required a higher dose of midazolam to achieve moderate sedation, suggesting a greater sedative requirement compared to the pethidine group. Nevertheless, the small difference in midazolam dosage is unlikely to have significantly contributed to the prolonged recovery time. These results suggest that the combination of midazolam and pethidine hydrochloride is a more suitable sedation method for outpatient EUS than the combination of midazolam and pentazocine.

Patients sometimes experience nausea or vomiting after undergoing EUS in the recovery room. Opioid analgesics can also cause nausea and vomiting.^28^ The analgesic effects of opioids are mediated by activation of three receptor subtypes: mu (μ), delta (δ), and kappa (κ).^29^, ^30^ Pethidine hydrochloride acts on the μ receptors. Pentazocine is an antagonist of μ receptors and a stimulator of κ receptors. In the present study, the frequencies of nausea or vomiting and readmission to the recovery room were markedly lower in the pethidine hydrochloride group than in the pentazocine group. None of the cases in the pethidine hydrochloride group complained of nausea or vomiting or were readmitted to the recovery room. It has been suggested that differences in receptor action may be associated with the frequency of side effects.^31^, ^32^

In the present study, we employed the hospital's own discharge criteria. Typical discharge criteria include the Aldrete scoring system and modified post‐anesthesia discharge scoring system. These are based on clinical criteria such as respiration, oxygen saturation, blood pressure, and level of consciousness.^33^, ^34^ However, these discharge criteria do not include the rest time after the completion of the examination, which makes them difficult to use in clinical practice. In many cases, the discharge criteria developed by each facility are used to determine whether a patient is ready for discharge.^35^ Our discharge criteria largely aligned with the Aldrete scoring system and modified post‐anesthesia discharge scoring system, and the minimum rest time after the completion of EUS was set at 45 min, which is considered a reasonable discharge criterion.

Propofol sedation may be better for diagnostic EUS than a combination of sedatives and analgesics.^14^, ^22^, ^23^ Propofol is a short‐acting sedative that potentiates gamma‐aminobutyric acid activity in the central nervous system. Midazolam, pethidine hydrochloride, and propofol all have an onset of action within approximately 5 min, but the durations of the effect are 15–80 min for midazolam and 60–180 min for pethidine hydrochloride, whereas that of propofol is 4–8 min.^14^ Sedation with propofol has similar rates of sedation‐related adverse effects, higher patient satisfaction, shorter time to sedation, and shorter recovery time than sedation with a combination of midazolam and pethidine hydrochloride for diagnostic EUS.^26^, ^36^, ^37^, ^38^ However, the use of propofol requires supervision by an anesthesiologist or a physician trained in anesthesia, making it difficult to use propofol in outpatients.^15^, ^39^, ^40^

This study has some limitations. First, this study is a retrospective single‐center observational study that does not include data on patient comorbidities or detailed patient backgrounds. Thus, further multicenter, large‐scale studies are required. Second, operator and patient satisfaction were not assessed. Therefore, it is difficult to evaluate whether the appropriate level of sedation was maintained during diagnostic EUS. Third, the median age of cases included in this study is 69 years, which is relatively young. Sedated endoscopy should be carefully discussed in older adult patients because of the increased risk of sedation‐related adverse events caused by the combination of sedatives and analgesics.^13^, ^15^, ^16^

In conclusion, using propensity score matching, this study revealed that the combination of midazolam and pethidine hydrochloride is a more favorable sedation approach than the combination of midazolam and pentazocine for diagnostic EUS in outpatients.

## CONFLICT OF INTEREST STATEMENT

The authors declare no conflict of interest.

## ETHICS STATEMENT

This study was approved by the Institutional Review Board of Osaka International Cancer Institute (approval number: 20117‐6) and was designed and executed in accordance with the ethical standards of the 1964 Declaration of Helsinki and its later amendments. Informed consent to participate in the study was waived in accordance with the Japanese government's Ethical Guidelines for Medical and Health Research Involving Human Subjects, which allow an opt‐out approach for studies based on existing, anonymized information that do not include human biological specimens.

## Supporting information

TABLE S1 Univariate and multivariate analysis of factors associated with readmission to the recovery room.

## Data Availability

Data supporting the findings of this study are available upon reasonable request from the corresponding author.
