# Supplementary material for: Comparison of sedation with pentazocine or pethidine hydrochloride for endoscopic ultrasonography in outpatients: A single‐center retrospective study
Source: DEN Open. 2024 Dec 31;5(1):e70048. doi: 10.1002/deo2.70048 (PMC11687558; doi:10.1002/deo2.70048)
Supplement: Supplementary file 1 — TABLE S1 Univariate and multivariate analysis of factors associated with readmission to the recovery room. [file DEO2-5-e70048-s001.docx]

**Supplementary Table 1**

Univariate and multivariate analysis of factors associated with readmission to the recovery room

|  | | Univariate analysis | | |  | Multivariate analysis | | |
| --- | --- | --- | --- | --- | --- | --- | --- | --- |
|  |  | OR | 95% CI | P-value |  | OR | 95% CI | P-value |
| Sex | Female | 4.489 | 1.947–10.352 | <0.001 |  | 8.220 | 3.482–19.404 | <0.001 |
| Age | >75 years | 1.653 | 0.800–3.417 | 0.175 |  | 2.035 | 0.943–4.391 | 0.070 |
| BMI | >25 kg/m^2^ | 0.723 | 0.313–1.672 | 0.435 |  |  |  |  |
| Procedure time | >25 min | 0.599 | 0.295–1.214 | 0.155 |  | 0.891 | 0.424–1.871 | 0.760 |
| Operator of EUS | trainee | 1.181 | 0.582–2.395 | 0.643 |  |  |  |  |
| Analgesics | Pentazocine | 11.733 | 4.117–33.439 | <0.001 |  | 17.682 | 6.066–51.542 | <0.001 |
| Total midazolam dose | >0.072 (mg/BW) | 0.844 | 0.430–1.656 | 0.621 |  |  |  |  |

OR, odds ratio; CI, confidence interval; BMI, body mass index; EUS, endoscopic ultrasonography; BW, body weight.

Statistical significance was set at *p*<0.05
